# Preferences for prenatal diagnosis of sickle‐cell disorder: A discrete choice experiment comparing potential service users and health‐care providers

**DOI:** 10.1111/hex.12568

**Published:** 2017-05-15

**Authors:** Melissa Hill, Eugene Oteng‐Ntim, Frida Forya, Mary Petrou, Stephen Morris, Lyn S. Chitty

**Affiliations:** ^1^ Genetics and Genomic Medicine UCL Great Ormond Street Institute of Child Health and Great Ormond Street Hospital for Children NHS Foundation Trust London UK; ^2^ Directorate of Women's Health Guy's and St Thomas’ Foundation Trust London UK; ^3^ King's College London London UK; ^4^ Fetal Medicine Unit University College London Hospitals NHS Foundation Trust London UK; ^5^ Institute of Women's Health University College London London UK; ^6^ Haemoglobinopathy Genetics Service University College London Hospitals NHS Foundation Trust London UK; ^7^ Department of Applied Health Research University College London London UK

**Keywords:** discrete choice experiment, non‐invasive prenatal diagnosis, sickle‐cell disorder

## Abstract

**Background:**

Non‐invasive prenatal diagnosis (NIPD) for sickle‐cell disorder (SCD) is moving closer to implementation and studies considering stakeholder preferences are required to underpin strategies for offering NIPD in clinical practice.

**Objective:**

Determine service user and provider preferences for key attributes of prenatal diagnostic tests for SCD and examine views on NIPD.

**Method:**

A questionnaire that includes a discrete choice experiment was used to determine the preferences of service users and providers for prenatal tests that varied across three attributes: accuracy, time of test and risk of miscarriage.

**Results:**

Adults who were carriers of SCD or affected with the condition (N=67) were recruited from haemoglobinopathy clinics at two maternity units. Health professionals, predominately midwives, who offer antenatal care (N=62) were recruited from one maternity unit. No miscarriage risk was a key driver of decision making for both service users and providers. Service providers placed greater emphasis on accuracy than service users. Current uptake of invasive tests was 63%, whilst predicted uptake of NIPD was 93.8%. Many service users (55.4%) and providers (52.5%) think pressure to have prenatal testing will increase when NIPD for SCD becomes available.

**Conclusions:**

There are clear differences between service users and health professionals’ preferences for prenatal tests for sickle‐cell disorder. The safety of NIPD is welcomed by parents and uptake is likely to be high. To promote informed choice, pretest counselling should be balanced and not exclusively focused on test safety. Counselling strategies that are sensitive to feelings of pressure to test will be essential.

## 1 BACKGROUND

Sickle‐cell disorders (SCD) affect the structure of haemoglobin and result in episodes of acute pain, chronic anaemia and progressive organ damage. Recent improvements in life expectancy for people affected with SCD have been attributed to earlier detection and improvements in comprehensive management.^1^ SCD is a relatively common condition, with approximately 12 000–15 000 affected individuals in the UK.^2^, ^3^ Whilst the condition is most common in people of African origin, SCD may occur in any ethnic group.^3^

SCD is caused by mutations in the β‐globin gene. Inheritance is autosomal recessive, so both parents must be carriers of a mutation for the baby to be at risk of SCD. In the UK, there is a universal neonatal screening programme for SCD and carrier screening is offered through an antenatal haemoglobinopathy screening programme that aims to offer screening by 10 weeks in pregnancy.^4^ Prenatal diagnosis of SCD is the most frequently requested prenatal test for a single gene disorder in the UK,^5^ and currently requires invasive testing (chorionic villus sampling or amniocentesis) which carry a small risk of miscarriage^6^, ^7^ and can only be carried out after 11 weeks in pregnancy. A safe alternative is on the horizon, with proof‐of‐concept studies showing that non‐invasive prenatal diagnosis (NIPD) based on analysis of cell‐free DNA (cfDNA) in maternal plasma may be possible for SCD.^8^ NIPD can be performed early in pregnancy (9‐10 weeks) and requires only a maternal blood sample, thereby removing the risk of procedure‐related miscarriage associated with invasive diagnostic tests. It is anticipated that NIPD for SCD will be diagnostic and there will be no need to confirm NIPD with invasive testing. As such, NIPD could be considered as a replacement for invasive testing.

It is important that research exploring views of NIPD is carried out alongside the technical development of these new tests to ensure that social and ethical issues are addressed and stakeholder needs are met. Previous research exploring views of NIPD for single gene disorders, including SCD, has been undertaken using qualitative interviews and focus groups with carriers of single gene disorders and health professionals.^9^, ^10^, ^11^ To expand this work, we have utilized a quantitative approach that includes a discrete choice experiment (DCE). DCEs allow us to look closely at people's decision‐making processes when making choices about prenatal testing. This is done by giving participants a series of hypothetical options for prenatal tests with differing attributes and asking them to choose between them. Analysing the choices, they make will identify the test attributes that are most important for decision making. We will also gain insights into people's willingness to trade one attribute for another. DCEs have been used to reveal preferences for screening and diagnostic tests for Down syndrome,^12^, ^13^, ^14^, ^15^, ^16^, ^17^ and we have also used a DCE to look at preferences for the prenatal diagnosis of cystic fibrosis.^18^ Here, we used a DCE to explore service user and health professional preferences for three key attributes of prenatal diagnostic tests for SCD: accuracy, time of test and risk of miscarriage. We also examined views on NIPD, including expected uptake. We tested two hypotheses:

1. Service users and health professionals will differ in their preferences for the three attributes of prenatal tests to be examined: safety, accuracy and time of testing;
2. Service users will value the safety afforded by NIPD and hypothetical uptake of the test will be high.

## 2 METHOD

### 2.1 Ethical approval

Ethical approval was obtained from a National Research Ethics Service Committee (10/H0714/3).

### 2.2 Recruitment

Two groups of participants were recruited: (i) service users: adults who were either carriers of SCD or affected with the condition, aged 18 or over and attending haemoglobinopathy antenatal clinics at either St Thomas's Hospital or University College London Hospital, and (ii) service providers: health professionals who deliver antenatal care and see women with pregnancies at risk of SCD at St Thomas’ Hospital.

Convenience sampling was used to participants to the study. For the service users, group potential participants were invited to anonymously complete the questionnaire whilst waiting for their clinical appointment. For the service provider group, potential participants were approached in person in their workplace and invited to anonymously complete a hard copy of the questionnaire.

### 2.3 Questionnaire design

The questionnaire had three components: (i) DCE choice sets, (ii) structured questions about prenatal testing and NIPD and (iii) demographic questions. Questionnaire design has been described previously, as the questionnaire used here was a modified version of the questionnaire used in our previous study looking at prenatal testing for cystic fibrosis.^18^ Briefly, design of the choice sets followed DCE guidelines^19^, ^20^, ^21^ and the attributes of safety, accuracy and time of test results were derived from focus groups with carriers of single gene disorders (SCD, cystic fibrosis and thalassaemia).^10^ The attributes and levels used in the DCE choice sets are presented in Table 1. There were eight choices in the DCE. One choice set served as an internal consistency check as one test was clearly better than the other. Participants had the choice of Test A, Test B or neither (Table 1). The structured questions comprised the following: ranking five attributes of prenatal tests (early testing, accuracy, financial cost, safety and comprehensive information) and a series of questions gathering views on prenatal testing for SCD which included two free‐text questions on benefits and concerns about NIPD. Demographic questions for service users covered age, gender, ethnicity, education and number of children. Demographic questions for health professionals covered job title, years in role, age and gender.

**Table 1:** Discrete choice experiment design

(A) Attributes and levels used in the discrete choice experiment	
Attribute	Levels
Accuracy	90%, 95%, 98, 100%
Time of results (gestation in weeks)	8, 10, 12, 14
Risk of miscarriage	Small risk (1%), No risk

(B) Example of a discrete choice experiment choice set		
Choice 1	Test A	Test B
Accuracy	95%	100%
Time of results	10 wk	12 wk
Risk of miscarriage	Small risk (1%)	No risk
Which test would you prefer (*tick one box only*)?		
Test A □	Test B □	Neither □

### 2.4 Analysis

DCE analysis followed published guidance.^19^, ^20^, ^21^ The DCE preference data were analysed using a conditional logit regression model as previously described.^18^ As the sign (+ or −) of the coefficients from the regression analysis shows the direction of the preference, we expected positive coefficients for accuracy and no miscarriage risk and a negative coefficient for timing, which would indicate a preference for earlier test. Notably, as the different attributes do not have the same unit of change, the absolute value of the coefficients cannot be directly compared. Consequently, to compare the preferences of service users and service providers, a common scale was created by calculating the marginal rates of substitution (MRS) as a ratio of the coefficients of two attributes.^20^ Descriptive statistics was used for the other components of the questionnaire. The software package Stata 12.0 (StataCorp USA) was used to perform all analyses.

## 3 RESULTS

### 3.1 Participants

Questionnaires were completed by 78 service users and 62 health professionals. Questionnaires were excluded if the internal consistency question was incorrect (patients n=7, health professionals n=0) or if the choice set was incomplete (patients n=4, and health professionals n=0). Ultimately, 67 service user and 62 health professional questionnaires were included in the analysis. The service user group included carriers of SCD (88.2%) and people affected with the condition (18.8%). The majority were female (89.4%). A small proportion had a child with SCD (16.4%). Health professionals were predominately midwives (88.1%) and female (91.8%). Demographic information for service users and providers is summarized in Tables 2 and 3.

**Table 2:** Health professional demographic data

	Total (n=62)
Age in years	
Mean (SD)	36.8 (9.15)
Gender	
Female	56 (91.8%)
Male	5 (8.2%)
Profession	
Midwife	42 (88.1%)
Consultant	4 (6.8%)
Specialist nurse	1 (1.7%)
Sonographer	2 (3.4%)
Years in profession	
≤5	29 (47.5%)
6‐15	23 (37.7%)
16‐25	9 (14.8%)

In some cases, numbers may not add up to total N due to missing data.

Percentages may not add up to 100 due to rounding.

**Table 3:** Service user demographic data

	Total (n=67)
Carrier status	
Carrier of sickle‐cell disorder	52 (81.2%)
Affected with sickle‐cell disorder	12 (18.8%)
Gender	
Female	59 (89.4%)
Male	7 (10.6%)
Age in years	
Mean (SD)	32.2 (5.46)
Ethnicity	
African/Caribbean	62 (92.5%)
Other	5 (7.5%)
Highest qualification	
No qualification	3 (4.7%)
High school	9 (14.1%)
Some college or other training	7 (10.9%)
Degree or equivalent	45 (70.3%)
Relationship status	
Married/In a relationship	54 (80.6%)
Separated/Divorced	5 (7.5%)
Widowed	1 (1.5%)
Single	7 (10.4%)
Religious faith	
Yes	62 (93.9%)
No	4 (6.1%)
Currently pregnant	
Yes	64 (97.0%)
No	2 (3.0%)
Number of children	
None	25 (37.9%)
1	22 (33.3%)
2	13 (19.7%)
3 or more	6 (9.1%)
Child with sickle‐cell disorder?	
Yes	9 (16.4%)
No	46 (83.6%)

In some cases, numbers may not add up to total N due to missing data.

Percentages may not add up to 100 due to rounding.

### 3.2 Regression results

Service users and providers prefer a test with greater accuracy, early testing and no risk of miscarriage (Table 4). The direction of the preferences matched our a priori expectations and supports the validity of the models. All coefficients were statistically significant for service providers. For service users, coefficients for accuracy and risk of miscarriage were significant, but time of results was not. Service providers placed greater value on test accuracy than service users, but no difference was seen for test safety or time of results. Analysis of participants’ willingness to trade between attributes was determined. For service users, 35 (52.2%) chose tests that carried no risk of miscarriage for all options, five (7.5%) chose tests with the highest accuracy for all options and one (1.5%) chose tests with the earliest time. For health professionals, 11 (17.7%) chose tests that carried no risk of miscarriage for all options, four (6.5%) chose tests with the highest accuracy for all options and none chose solely based on test timing.

**Table 4:** Conditional logit regression comparing service users and health professionals

	Accuracy	Time of results	No risk of miscarriage
	Coefficient (95% CI)	Coefficient (95% CI)	Coefficient (95% CI)
Service users (n=67)^a^	0.114 (0.074 to 0.154)	−0.047^a^ (−0.108 to −0.013)	1.760 (1.509 to 2.011)
Health professionals (n=62)^b^	0.304 (0.243 to 0.365)	−0.130 (−0.192 to −0.068)	1.768 (1.422 to 2.113)
Difference (*P*‐value)	<.0001	.0661	.9717

CI‐confidence interval.

a Number of observations=1605; pseudo‐*R* ^2^=.4438.

b Number of observations=1488; pseudo‐*R* ^2^=.5161.

Coefficient not significant. All other coefficients significant *P*<.0001.

### 3.3 Marginal rates of substitution

The MRS demonstrates the strong preference held by both service users and health professionals for a test with no risk of miscarriage. Notably, service users were prepared to wait longer and accept lower accuracy compared to service providers for a test that had no risk of miscarriage (Table S1).

### 3.4 Ranking of attributes

Participants ranked the importance of five attributes of prenatal tests: safety, early testing, accuracy, financial cost and comprehensive information (Tables S2 and S3). Safety was ranked highest by service users (43.2%) followed by full information (21.6%). Accuracy was ranked highest by health professionals (45.6%) followed by safety (42.1%). Cost was ranked lowest by both service users (86.5%) and health professionals (57.9%).

### 3.5 Views on prenatal testing and the introduction of NIPD

When service users were asked whether they would have invasive testing for SCD, 63.1% said they have had or are likely to have invasive testing, with the most common reasons for testing being to “help make a decision about whether or not to continue the pregnancy” (47.4%) and “to plan and prepare for the possible birth of a baby with SCD” (36.8%) (Table 5). More than half (53.8%) said they would never have an invasive test because of the risk of miscarriage. When asked whether they would have NIPD for SCD if it was available, 93.8% indicated that they would have NIPD. The majority of service users (73.4%) said that they would be prepared to pay for NIPD.

**Table 5:** Service user uptake of prenatal testing

	Total (n=67)
Have had/likely to have an invasive test for SCD	
Strongly agree/Agree	41 (63.1%)
Strongly disagree/Disagree	24 (36.9%)
Reason for choosing to have a diagnostic test	
To plan and prepare for the possible birth of a baby with SCD	14 (36.8%)
To help make a decision about whether or not to continue the pregnancy	18 (47.4%)
Because my family or my partner would want me to	1 (2.6%)
Because it is offered as part of the antenatal service	5 (13.4%)
Other	0 (0%)
Would never have an invasive test because would not consider termination of pregnancy	
Strongly agree/Agree	28 (45.2%)
Strongly disagree/Disagree	34 (54.8%)
Would never have an invasive test because of the risk of miscarriage	
Strongly agree/Agree	35 (53.8%)
Strongly disagree/Disagree	30 (46.2%)
Would have NIPD if available	
Strongly agree/Agree	60 (93.8%)
Strongly disagree/Disagree	4 (6.3%)
Willingness to pay for NIPD	
≤£50	37 (57.8%)
£100‐200	8 (12.5%)
≥£200	2 (3.1%)
Not prepared to pay	17 (26.6%)

All participants were asked their views on pressure to have prenatal testing for SCD and whether they felt that offering NIPD would increase feelings of pressure to have a diagnostic test (Table 6). Approximately half of both the service users (55.4%) and health professionals (52.5%) thought that pressure to have prenatal testing would increase if NIPD for SCD became available. Half of the service providers (50%) and more than half of service users (57.1%) felt that there was already pressure to have prenatal testing for SCD. The most frequently cited source of pressure was health professionals for both service users (46.5%) and providers (36.9%).

**Table 6:** Views on pressure to have prenatal testing

	Service users (n=67)	Health professionals (n=62)
There is pressure on women at risk of having a child with SCD to have a diagnostic test in pregnancy		
Strongly agree/Agree	36 (57.1%)	30 (50.0%)
Strongly disagree/Disagree	27 (42.9%)	30 (50.0%)
If you agree, where do you think this pressure comes from^a^		
Partner	6 (14.0%)	6 (9.2%)
Family members	9 (20.9%)	15 (23.1%)
Health professionals	20 (46.5%)	24 (36.9%)
Society in general	5 (11.6%)	10 (15.4%)
Your cultural or religious community	2 (4.9%)	10 (15.4%)
Other	1 (2.3%)	0 (0%)
The availability of NIPD will increase pressure to have prenatal testing		
Strongly agree/Agree	31 (55.4%)	31 (52.5%)
Strongly disagree/Disagree	25 (44.6%)	28 (47.5%)

a Participants could choose multiple responses.

Forty‐three (64.2%) service users responded to the question on what they saw as the benefits of NIPD. These primarily fell into three categories: no miscarriage risk (n=21); decision making, knowledge and preparation (n=17); and simpler and less stressful testing (n=6). Fifty‐nine (95.2%) health professionals described potential benefits of NIPD, with the majority mentioning decision making, knowledge and preparation (n=46); no miscarriage risk (n=15); and early diagnosis (n=11). Forty‐five service users reported that they had no concerns (67.2%) and five said they had concerns but did not elaborate. Twenty‐nine (46.8%) health professionals reported that they had no concerns. Twenty‐six (41.9%) health professionals described concerns, with the most common being increased pressure to test and terminate (n=11), availability of appropriate counselling (n=7), accuracy (n=6) and costs (n=4).

To address the question of whether health professionals thought NIPD should only be offered if it would change pregnancy management, a question was included asking whether NIPD should only be offered to women considering termination of pregnancy. Only four (6.6%) strongly agreed or agreed compared to 57 (93.4%) who strongly disagreed or disagreed.

## 4 DISCUSSION

The aim of this study was to determine the preferences held by potential service users and providers for prenatal tests for SCD. Test safety was an important driver of decision making for both groups. Differences were evident in the emphasis placed on test accuracy, with service providers placing greater emphasis on this attribute than service users. It was clear that service users were prepared to wait longer and accept lower accuracy than service providers for a test with no risk of miscarriage. In addition, over half of the service users chose tests based only on test safety and did not consider the other attributes. Differences between health professionals and potential service users have been seen in other DCE studies comparing non‐invasive and invasive tests for cystic fibrosis^18^ and also for Down syndrome.^15^, ^16^, ^17^ Our findings draw attention to the need for health professionals to be mindful that their own views on what is important when making decisions about prenatal testing may differ from those held by parents. Moreover, the emphasis on test safety when making decisions about prenatal testing points towards the possibility that parents may not make an informed choice as other attributes may not be considered. Consequently, it is important that health professionals discuss the benefits and limitations of NIPD, taking care not to focus solely on discussing the safety of the test. Alternatives to NIPD should also be discussed in a balanced way and must include the option of not having testing. Formal consent processes could also be used to emphasize that NIPD is a diagnostic test that needs careful consideration.^11^, ^22^, ^23^

Previous research looking at NIPD for single gene disorders found that both potential service users and providers thought that the ease and safety of NIPD may put couples under increased pressure to have prenatal testing.^9^, ^10^, ^18^ Similarly, we found that approximately half of service users (55.4%) and providers (52.5%) felt that offering NIPD would increase pressure to have a prenatal test to diagnose SCD. Moreover, the DCE results demonstrate that test safety is a key driver of decisions for parents considering prenatal testing. Service providers offering NIPD for SCD need to be aware that offering NIPD may create feelings of pressure to have diagnostic testing. The most commonly reported source of pressure was health professionals and it may be that feelings of pressure arise from how the test is presented or because it has been offered by a “trusted” health professional.^24^ This is particularly pertinent in offering NIPD for SCD as previous research with women offered carrier testing for SCD during pregnancy indicated that the woman in the study accepted that health professionals had authoritative knowledge and the fact that screening was offered implied that having a baby with SCD would not be a good outcome.^25^ Careful pretest counselling is needed to avoid parents feeling pressured to test and to allow them to make choices in keeping with their personal beliefs and values.

We found that willingness to have NIPD was high, with over 90% of service users saying they would have NIPD for SCD if it was available compared to 63% who said they would currently choose invasive testing. In addition, approximately 50% reported that risk of miscarriage was a barrier to invasive prenatal testing. These findings are similar to those of other studies exploring the potential uptake of NIPD for single gene disorders.^9^, ^10^, ^18^, ^26^ Previous research has highlighted the difficulties in achieving timely partner testing within the UK Sickle Cell and Thalassaemia Screening Programme.^27^ In 2013‐2014, 15281 pregnant women were screen positive and 64% of partners had carrier screening.^28^ It is possible that the number of women opting for NIPD for SCD may increase further because women whose partners decline carrier testing may find NIPD more acceptable than invasive testing when the exact risk of the child inheriting SCD is unknown.^10^, ^11^ Overall, it appears that uptake of NIPD for single gene disorders will be high and many couples who would not consider invasive testing due to the risk of miscarriage would have NIPD.

The changes to the population accepting the offer of prenatal testing need to be considered in our approaches to counselling for NIPD for SCD. Most notably, many couples who would not previously have had prenatal testing may take up NIPD, and as a result may find themselves faced with a decision about termination of pregnancy. This issue is particularly important in the setting of prenatal testing for SCD where in the UK and other countries a prenatal carrier screening programme is in place and there are existing concerns about informed consent processes^29^ and carrier screening being presented as routine.^30^ Without careful pretest counselling, the addition of NIPD to the care pathway has the potential to undermine informed consent as women newly identified as carriers of SCD may see NIPD as routine next step following a positive carrier screening result.^31^ As such, it will be especially important to allow sufficient time in pretest counselling to talk through the impact of having NIPD which includes a discussion around the implications of the possible test results that is guided by the parents values and preferences.^32^ It must be made clear to parents that accepting a “simple blood test” could lead to a decision about whether to continue or terminate the pregnancy. Individualized support through post‐test counselling to assist decisions about next steps will be essential.

The cost of NIPD for SCD will need to be considered in strategies to implement this test in the NHS. The potential cost of NIPD relative to the current invasive testing pathway has been explored and NIPD for SCD using current approaches was estimated to cost £1210, which was £190 more than invasive testing. Moreover, the anticipated high uptake of NIPD we found in this study (approximately 95% compared to 65% for invasive testing) would result in an incremental cost of NIPD over invasive testing of £48,635 per 100 pregnancies at risk of SCD. As the increased uptake will include parents who would want to have NIPD for information only and would not consider termination of pregnancy, there is a need to address the issue of whether it is appropriate to direct resources to test when pregnancy management would not change. Consideration of this question must include the benefits of the information for early reassurance or for planning and preparation if the baby is found to be affected by SCD.^31^ In the study, we report here only a very small proportion of health professionals (6.6%), when asked, thought NIPD should only be offered if it would change pregnancy management.

### 4.1 LIMITATIONS

Several limitations of our study may mean that our findings are not widely generalizable. Recruitment was only conducted at two centres for service users and only one centre for service providers and both centres were located in central London. As this was not a random sample and convenience sampling was used, it is possible that sampling bias will limit the generalizability of our results. In addition, participants were self‐selected and there may be responder bias towards people with strong pre‐existing views on NIPD. Another limitation of the study was that the numbers of participants recruited were not sufficient to allow subgroup comparisons. In an equivalent study looking at NIPD for cystic fibrosis, we found there were differences in preferences between people affected with the condition and those who were carriers.^18^ In addition, only a small number of men were recruited and their viewpoints may differ to those of women. The health professional group primarily comprised midwives and health professionals from other training backgrounds may have different preferences. In future studies, it would be useful to seek the views of other professionals and include people with both obstetrics and genetics backgrounds. As this is a stated preference study, it is possible that the choices made by participants may not reflect real‐life decisions. The DCE only included three attributes of prenatal tests, and in reality, many attributes are considered when making decisions about testing. In addition, the DCE design does not address why these choices have been made or give insight into how the tests were perceived. Another important limitation is that participants’ reported willingness to have NIPD is hypothetical and may not reflect uptake when the test enters clinical practice which has been seen in other studies looking at uptake of genetic testing.^33^, ^34^, ^35^

## 5 CONCLUSIONS

When making decisions about prenatal testing for SCD, potential service users and providers do not place the same emphasis on the test attributes. It is likely that the safety of NIPD will be welcomed by parents and uptake will be high. It is therefore important that pretest counselling is balanced and not predominately focused on the safety of NIPD. Care must also be taken to minimize feelings of pressure to have NIPD. Considerations for implementation need to include current carrier screening and prenatal diagnosis pathways. Offering NIPD as a next step in the current pathway could create pressure to test at a time when news of carrier status is still being processed and decisions need to be made quickly about next steps. Thorough pre‐ and post‐test counselling will be essential and NIPD should be offered by health professionals specifically trained in counselling for prenatal testing for SCD.

## CONFLICTS OF INTEREST

The authors declare no conflict of interest.

## References

[1] Rees DC , Williams TN , Gladwin MT . Sickle‐cell disease. Lancet. 2010;376:2018‐2031. 21131035 10.1016/S0140-6736(10)61029-X

[2] Streetly A , Latinovic R , Hall K , Henthorn J . Implementation of universal newborn bloodspot screening for sickle cell disease and other clinically significant haemoglobinopathies in England: screening results for 2005‐7. J Clin Pathol. 2009;62:26‐30. 19103854 10.1136/jcp.2008.058859 PMC2603283

[3] Piel FB , Patil AP , Howes RE , et al. Global epidemiology of sickle haemoglobin in neonates: a contemporary geostatistical model‐based map and population estimates. Lancet. 2013;381:142‐151. 23103089 10.1016/S0140-6736(12)61229-X PMC3547249

[4] NHS Sickle Cell and Thalassaemia Programme . Standards for the linked antenatal and newborn screening program. https://www.gov.uk/guidance/sickle-cell-and-thalassaemia-screening-programme-overview. Accessed March 16, 2016

[5] Association of Clinical Genetics Science Quality Subcommittee . Association of Clinical Genetics Science Audit of Data 2012‐13. http://www.acgs.uk.com/media/822585/acgsaudit12_13_final2.pdf Accessed September 20, 2015.

[6] Tabor A , Alfirevic Z . Update on procedure‐related risks for prenatal diagnosis techniques. Fetal Diagn Ther. 2010;27:1‐7. 20051662 10.1159/000271995

[7] Akolekar R , Beta J , Picciarelli G , Ogilvie C , D'Antonio F . Procedure‐related risk of miscarriage following amniocentesis and chorionic villus sampling: a systematic review and meta‐analysis. Ultrasound Obstet Gynecol. 2015;45:16‐26. 25042845 10.1002/uog.14636

[8] Barrett AN , McDonnell TC , Chan KC , Chitty LS . Digital PCR analysis of maternal plasma for noninvasive detection of sickle cell anemia. Clin Chem. 2012;58:1026‐1032. 22451622 10.1373/clinchem.2011.178939

[9] Hill M , Karunaratna M , Lewis C , Forya F , Chitty L . Views and preferences for the implementation of non‐invasive prenatal diagnosis for single gene disorders from health professionals in the United Kingdom. Am J Med Genet A. 2013;161A:1612‐1618. 23696422 10.1002/ajmg.a.35972

[10] Hill M , Compton C , Karunaratna M , Lewis C , Chitty L . Client views and attitudes to non‐invasive prenatal diagnosis for sickle cell disease, thalassaemia and cystic fibrosis. J Genet Couns. 2014;23:1012‐1021. 24788196 10.1007/s10897-014-9725-4

[11] Skirton H , Goldsmith L , Chitty LS . An easy test but a hard decision: ethical issues concerning non‐invasive prenatal testing for autosomal recessive disorders. Eur J Hum Genet. 2015;23:1004‐1009. 25351779 10.1038/ejhg.2014.238 PMC4795110

[12] Bishop A , Marteau T , Armstrong D , et al. Women and health professional's preferences for Down's Syndrome screening tests: a conjoint analysis study. BJOG. 2004;111:775‐779. 15270923 10.1111/j.1471-0528.2004.00197.x

[13] Ryan M , Diack J , Watson V , Smith N . Rapid prenatal diagnostic testing for Down syndrome only or longer wait for full karyotype: the views of pregnant women. Prenat Diagn. 2005;25:1206‐1211. 16353286 10.1002/pd.1309

[14] Lewis SM , Cullinane FN , Bishop AJ , et al. A comparison of Australian and UK obstetricians’ and midwives’ preferences for screening tests for Down syndrome. Prenat Diagn. 2006;26:60‐66. 16378328 10.1002/pd.1357

[15] Hill M , Fisher J , Chitty LS , Morris S . Women's and health professionals’ preferences for prenatal tests for Down syndrome: a discrete choice experiment to contrast noninvasive prenatal diagnosis with current invasive tests. Genet Med. 2012;14:905‐913. 22935718 10.1038/gim.2012.68

[16] Beulen L , Grutters JP , Faas BH , et al. Women's and healthcare professionals’ preferences for prenatal testing: a discrete choice experiment. Prenat Diagn. 2015;35:549‐557. 25644120 10.1002/pd.4571

[17] Hill M , Johnson JA , Langlois S , et al. Preferences for prenatal tests for Down syndrome: an international comparison of the views of pregnant women and health professionals. Eur J Hum Genet. 2016;24:968‐975. 26577044 10.1038/ejhg.2015.249 PMC5070900

[18] Hill M , Suri R , Nash E , Morris S , Chitty LS . Preferences for prenatal tests for cystic fibrosis: a discrete choice experiment to compare the views of adult patients, carriers of cystic fibrosis and health professionals. J Clin Med. 2014;3:176‐190. 26237256 10.3390/jcm3010176 PMC4449661

[19] Ryan M , Gerard K , Amaya‐Amaya M , editors. Using discrete choice experiments to value health and health care. Dordrecht, The Netherlands: Springer; 2008.

[20] Lancsar E , Louviere J . Conducting discrete choice experiments to inform healthcare decision making: a user's guide. Pharmacoeconomics. 2008;26:661‐677. 18620460 10.2165/00019053-200826080-00004

[21] Bridges JF , Hauber AB , Marshall D , et al. Conjoint analysis applications in health–a checklist: a report of the ISPOR Good Research Practices for Conjoint Analysis Task Force. Value Health. 2011;14:403‐413. 21669364 10.1016/j.jval.2010.11.013

[22] Lewis C , Hill M , Silcock C , Daley R , Chitty L . Non‐invasive prenatal testing for trisomy 21: a cross‐sectional survey of service users’ views and likely uptake. BJOG. 2014;121:582‐594. 24433394 10.1111/1471-0528.12579

[23] Farrell RM , Agatisa PK , Nutter B . What women want: lead considerations for current and future applications of noninvasive prenatal testing in prenatal care. Birth. 2014;41:276‐282. 24825739 10.1111/birt.12113 PMC4195446

[24] Lewis C , Silcock C , Chitty LS . Non‐invasive prenatal testing for Down's syndrome: pregnant women's views and likely uptake. Public Health Genomics. 2013;16:223‐232. 23886854 10.1159/000353523

[25] Tsianakas V , Atkin K , Calnan MW , Dormandy E , Marteau TM . Offering antenatal sickle cell and thalassaemia screening to pregnant women in primary care: a qualitative study of women's experiences and expectations of participation. Health Expect. 2012;15:115‐125. 21366810 10.1111/j.1369-7625.2011.00669.x PMC5060612

[26] Lewis C , Hill M , Chitty LS . Non‐invasive prenatal diagnosis for single gene disorders: experience of patients. Clin Genet. 2014;85:336‐342. 23631435 10.1111/cge.12179

[27] Dormandy E , Gulliford M , Bryan S , et al. Effectiveness of earlier antenatal screening for sickle cell disease and thalassaemia in primary care: cluster randomised trial. BMJ. 2010;341:c5132. 20923841 10.1136/bmj.c5132 PMC2950261

[28] Public Health England . NHS Screening Programmes in England. https://wwwgovuk/government/uploads/system/uploads/attachment_data/file/446648/Screening_in_England_2013-14_final_webpdf Accessed March 16, 2016.

[29] Locock L , Kai J . Parents’ experiences of universal screening for haemoglobin disorders: implications for practice in a new genetics era. Br J Gen Pract. 2008;58:161‐168. 18318970 10.3399/bjgp08X277276 PMC2249791

[30] Tsianakas V , Calnan M , Atkin K , Dormandy E , Marteau TM . Offering antenatal sickle cell and thalassaemia screening to pregnant women in primary care: a qualitative study of GPs’ experiences. Br J Gen Pract. 2010;60:822‐828. 21062549 10.3399/bjgp10X532602 PMC2965967

[31] Deans Z , Hill M , Chitty LS , Lewis C . Non‐invasive prenatal testing for single gene disorders: exploring the ethics. Eur J Hum Genet. 2012;21:713‐718. 23188047 10.1038/ejhg.2012.250 PMC3722948

[32] Hodgson J , Spriggs M . A practical account of autonomy: why genetic counseling is especially well suited to the facilitation of informed autonomous decision making. Eur J Hum Genet. 2005;14:89‐97. 10.1007/s10897-005-4067-x 15959640

[33] Sanderson SC , O'Neill SC , Bastian LA , Bepler G , McBride CM . What can interest tell us about uptake of genetic testing? Intention and behavior amongst smokers related to patients with lung cancer. Public Health Genomics. 2010;13:116‐124. 19556750 10.1159/000226595 PMC3696369

[34] Lerman C , Croyle RT , Tercyak KP , Hamann H . Genetic testing: psychological aspects and implications. J Consult Clin Psychol. 2002;70:784‐797. 12090383 10.1037//0022-006x.70.3.784

[35] Ropka ME , Wenzel J , Phillips EK , Siadaty M , Philbrick JT . Uptake rates for breast cancer genetic testing: a systematic review. Epidemiol Biomarkers Prev. 2006;15:840‐855. 10.1158/1055-9965.EPI-05-0002 16702359

