# Supplementary material for: Preferences for prenatal diagnosis of sickle‐cell disorder: A discrete choice experiment comparing potential service users and health‐care providers
Source: Health Expect. 2017 May 15;20(6):1289–95. doi: 10.1111/hex.12568 (PMC5689222; doi:10.1111/hex.12568)
Supplement: Supplementary file 1 [file HEX-20-1289-s001.docx]

**Supplementary Material**

**Table 1:** Marginal rates of substitution

|  | **Number of weeks respondents  are prepared to wait** | | **Reduction in accuracy (%) respondents  are prepared to accept** | |
| --- | --- | --- | --- | --- |
|  | Service users | Health professionals | Service users | Health professionals |
| Test with no risk of miscarriage | 37.45 (1.760/-0.047) | 13.60 (1.768/-0.130) | 12.22 (1.760/0.114) | 5.82 (1.768/0.304) |
| Test with 5% greater accuracy | 15.32 (0.114/-0.047 x5) | 11.69 (0.304/-0.130 x5) | - | - |
| Early Test | - | - | 0.33 (-0.047/0.114) | 0.43 (-0.130/0.304) |

**Table 2:** Ranking of test attributes – service users

| Attribute | **Ranking (%)*** | | | | |
| --- | --- | --- | --- | --- | --- |
|  | **1** | **2** | **3** | **4** | **5** |
| Early test | 16.2 | 13.5 | 27.0 | 25.1 | 8.1 |
| Accuracy | 16.2 | 40.4 | 29.7 | 13.5 | 0 |
| Cost | 2.7 | 0 | 2.7 | 8.1 | 86.5 |
| Safety | 43.2 | 24.3 | 18.9 | 13.5 | 0 |
| Full information | 21.6 | 21.6 | 21.6 | 29.7 | 5.4 |

* n = 37

**Table 3:** Ranking of test attributes – health professionals

| Attribute | **Ranking (%)*** | | | | |
| --- | --- | --- | --- | --- | --- |
|  | **1** | **2** | **3** | **4** | **5** |
| Early test | 7.0 | 19.3 | 38.6 | 28.1 | 7.0 |
| Accuracy | 45.6 | 36.8 | 12.3 | 5.2 | 0 |
| Cost | 1.8 | 1.8 | 10.5 | 28.1 | 57.9 |
| Safety | 42.1 | 33.3 | 14.0 | 7.0 | 3.5 |
| Full information | 3.5 | 8.8 | 24.6 | 31.6 | 31.6 |

* n = 57
